# Genomic, genetic and structural analysis of pyoverdine-mediated iron acquisition in the plant growth-promoting bacterium *Pseudomonas fluorescens *SBW25

**DOI:** 10.1186/1471-2180-8-7

**Published:** 2008-01-14

**Authors:** Christina D Moon, Xue-Xian Zhang, Sandra Matthijs, Mathias Schäfer, Herbert Budzikiewicz, Paul B Rainey

**Affiliations:** 1 Department of Plant Sciences, University of Oxford, South Parks Rd, Oxford OX1 3RB, UK; 2 School of Biological Sciences, University of Auckland, Private Bag 92019, Auckland, New Zealand; 3 Laboratory of Microbial Interactions, Department of Molecular and Cellular Interactions, Flanders Interuniversity Institute for Biotechnology, Vrije Universiteit Brussel, Building E, Pleinlaan 2, Brussels, Belgium; 4 Institut für Organische Chemie der Universität zu Köln, Greinstr. 4, 50939, Köln, Germany; 5 AgResearch Limited, Grasslands Research Centre, Private Bag 11008, Palmerston North, New Zealand; 6 Institute of Molecular Biosciences and New Zealand Institute for Advanced Study, Massey University, Private Bag 102904, Auckland, New Zealand

## Abstract

**Background:**

Pyoverdines (PVDs) are high affinity siderophores, for which the molecular mechanisms of biosynthesis, uptake and regulation have been extensively studied in *Pseudomonas aeruginosa *PAO1. However, the extent to which this regulatory model applies to other pseudomonads is unknown. Here, we describe the results of a genomic, genetic and structural analysis of pyoverdine-mediated iron uptake by the plant growth-promoting bacterium *P. fluorescens *SBW25.

**Results:**

*In silico *analysis of the complete, but un-annotated, SBW25 genome sequence identified 31 genes putatively involved in PVD biosynthesis, transport or regulation, which are distributed across seven different regions of the genome. PVD gene iron-responsiveness was tested using '*lacZ *fusions to five PVD loci, representative of structural and regulatory genes. Transcription of all fusions increased in response to iron starvation. *In silico *analyses suggested that regulation of *fpvR *(which is predicted to encode a cytoplasmic membrane-spanning anti-sigma factor) may be unique. Transcriptional assays using gene expression constructs showed that *fpvR *is positively regulated by FpvI (an extracytoplasmic family (ECF) sigma factor), and not directly by the ferric uptake regulator (Fur) as for PAO1. Deletion of *pvdL*, encoding a predicted non-ribosomal peptide synthetase (NRPS) involved in PVD chromophore biosynthesis confirmed the necessity of PvdL for PVD production and for normal growth in iron-limited media. Structural analysis of the SBW25 PVD shows a partly cyclic seven residue peptide backbone, identical to that of *P. fluorescens *ATCC13525. At least 24 putative siderophore receptor genes are present in the SBW25 genome enabling the bacterium to utilize 19 structurally distinct PVDs from 25 different *Pseudomonas *isolates.

**Conclusion:**

The genome of *P. fluorescens *SBW25 contains an extensively dispersed set of PVD genes in comparison to other sequenced *Pseudomonas *strains. The PAO1 PVD regulatory model, which involves a branched Fpv signaling pathway, is generally conserved in SBW25, however there is a significant difference in *fpvR *regulation. SBW25 produces PVD with a partly cyclic seven amino acid residue backbone, and is able to utilize a wide variety of exogenous PVDs.

## Background

Iron is a common co-factor for redox-dependent enzymes and an essential element for almost all living organisms. In nature iron is abundant; however, in aerobic environments and under general physiological conditions, iron typically exists in the insoluble ferric (Fe^3+^) form, thus rendering acquisition by organisms difficult. Under conditions of iron limitation, many bacteria produce and secrete low molecular weight ferric-specific ligands known as siderophores (for review, see [1]). The ferric-siderophores deliver iron to the cell *via *specific receptor and transport systems (for review, see [2]).

Collectively, *Pseudomonas *spp. produce a wide variety of siderophores [3], the most complex and common of which are pyoverdines (PVDs) [4]. PVDs contain a peptide moiety, usually between 6–12 amino acids in length, and a dihydroxyquinoline chromophore moiety [4], which gives PVD its characteristic yellow-green fluorescent appearance. PVD-mediated iron uptake processes have been extensively characterized in *Pseudomonas aeruginosa *PAO1, where key regulatory and structural proteins have been identified, and regulatory mechanisms have been elucidated [5-9].

In *P. aeruginosa *PAO1, the primary level of PVD regulation involves the ferric uptake regulator (Fur), which, upon interaction with ferrous iron (Fe^2+^), binds to a specific DNA sequence (the Fur-box) in the promoter region of certain iron-regulated genes and blocks transcription. Under iron-deplete conditions (no Fe^2+ ^available to interact with Fur) Fur-dependent repression is relieved [6,10]. PAO1 PVD genes that possess Fur boxes, which have been demonstrated to bind Fur *in vitro *[5,11], are *pvdS *and *fpvI*, which encode extracytoplasmic family (ECF) sigma factors, and *fpvR*, an inner membrane-spanning anti-sigma factor. The alternative sigma factor, PvdS, when associated with core RNA polymerase, binds iron-starvation (IS) box motifs [12] and directs the expression of a suite of genes involved in the synthesis of PVD [7,13]. The activity of PvdS is regulated by a transmembrane signaling system that comprises of FpvR and FpvA, an outer membrane PVD receptor, and is primarily mediated by PVD [5]. More recently, a second ECF sigma factor (FpvI) was found to direct transcription of *fpvA *[5,14]. Like PvdS, FpvI is also under the direct regulation of FpvR and forms a second divergent branch of the signaling pathway.

Many plant-associated pseudomonads are known to produce PVDs, including the plant pathogen, *P. syringae *and the saprophytes, *P. putida *and *P. fluorescens*. The importance of siderophores for rhizosphere colonization by *P. putida *has been previously reported [15,16]. However, little is known about the molecular mechanisms of PVD production and regulation in these organisms.

Here we report a genetic characterization of genes for PVD production in a plant growth-promoting bacterium, *Pseudomonas fluorescens *SBW25. SBW25 was originally isolated from field-grown sugar beet [17] and its biocontrol activity against the soil-borne pathogen, *Pythium ultimum*, is related to its considerable plant colonization ability [18]. The present investigation was prompted by initial findings that three genes implicated in iron-acquisition processes were up-regulated on plant surfaces, as revealed by *in vivo *expression technology (IVET) analysis [19,20]. Two genes had significant homology to putative siderophore receptor genes, and one gene appeared to be homologous to PAO1 *pvdL*, which encodes a non-ribosomal peptide synthetase (NRPS) involved in PVD biosynthesis [21]. These findings suggested a significant role for iron uptake during seedling colonization.

Our work began with the un-annotated whole genome sequence of SBW25 [22]. Based initially on an analysis of this sequence, 31 genes were identified with predicted roles in PVD biosynthesis, transport and regulation, for which the regulation of a subset of these was investigated by genetic analysis using chromosomally-integrated '*lacZ *fusions and gene expression constructs. The results indicate a not previously realized mechanism of *fpvR *regulation. Moreover, we determine the chemical structure of SBW25 PVD and examine the biological role of PVD *via *a siderophore-deficient mutant. The ability of SBW25 to utilize a panel of structurally distinct exogenous PVDs was also assessed.

## Results and Discussion

### Genomic analysis of PVD genes in *P. fluorescens *SBW25

**Figure 1:**
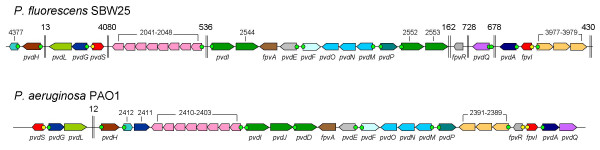
**Comparison of the arrangement of PVD genes in *P. fluorescens *SBW25 and *Pseudomonas aeruginosa *PAO1**. PAO1 PVD genes are annotated according to [23], and gene names, if given, are shown beneath genes, otherwise PA gene numbers are shown above. PVD gene homologues in SBW25 were identified by BLAST searches, and Pflu gene numbers [22] are shown above genes, if names are not given. Yellow and green dots preceding genes represent sequences that are highly consistent with the Fur-box (5'-GATAATGATAATCATTATC-3') [11], and IS-box (5'-TAAAT-N_16_-CGT-3') [12, 23, 41] consensus sequences, respectively. Gene sizes are not drawn to linear scale, and double vertical lines represent intervening DNA (sizes in kb shown above). Figure adapted from Ravel & Cornelis (2003).

Interrogation of the complete genome sequence of SBW25 using genes known to be involved in PVD-mediated iron acquisition in *P. aeruginosa *PAO1 [23] revealed 31 homologues with predicted roles in PVD biosynthesis, transport and regulation. The results are summarized in Figure 1 and Additional file 1.

SBW25 homologues were identified for all PAO1 PVD genes except PA2411, which encodes a thioesterase, and *pvdJ *and *pvdD*, which encode NRPSs involved in biosynthesis of the PVD peptide backbone. In PAO1, PA2411 is not essential for PVD production, and is possibly redundant since *pvdG *also encodes a thioesterase [23]. Although homologues of *pvdJ *and *pvdD *were not identified in SBW25, three additional NRPS genes were identified: two downstream of *pvdP *(Pflu2552 and Pflu2553) and one downstream of *pvdI *(Pflu2544; Figure 1). Differences in the sequences and organization of these NRPS genes likely reflect differences between the structures of the PVDs produced by SBW25 and PAO1 (discussed below).

The PVD genes of SBW25 are distributed across seven different loci within the genome (Figure 1), which is the most widespread distribution of PVD genes in a genomic comparison among four various sequenced *Pseudomonas *spp. [23]. This distribution is in stark contrast to that of *P. syringae *where PVD genes form one large cluster [23]. In *P. aeruginosa *PAO1 the PVD genes are confined to two loci separated by just 11.5 kb (Figure 1), which appears to be typical of *P. aeruginosa *isolates in general, where almost all PVD genes are located within the same region of the genome [24]. PVD gene homologues in *P. putida *KT2440 and *P. fluorescens *strains Pf0–1 [22] and Pf-5 [25] are distributed across three loci.

Despite the overall differences in the genomic organization of PVD genes between SBW25 and PAO1, genes within each of the seven SBW25 PVD clusters are largely syntenous with those in PAO1. There are, however, two exceptions: *pvdQ *and *fpvR*, which in SBW25 are found in two non-adjacent regions of the genome and separated from all other PVD genes. The isolated position of *fpvR *(especially in relation to *fpvI *– see Figure 1), is particularly unusual: the genes for ECF sigma factors and their cognate anti-sigma factors are typically adjacent, and usually co-regulated by Fur [2,26,27]. The gene arrangement in SBW25 suggests that *fpvR *and *fpvI *might be under separate regulatory control – a notion given credibility by the fact that the promoter sequence of *fpvR *in SBW25 does not have a recognizable Fur-box motif. Interestingly, in place of the expected Fur-box motif is an IS-box motif, albeit one in which the last three bases of the consensus sequence are in reverse order (TAAAT-N_16_-TGC, rather than TAAAT-N_16_-CGT). The possibility that in SBW25 *fpvR *might be regulated by PvdS led to further investigation (described below).

### Expression of putative PVD genes in response to iron starvation

**Figure 2:**
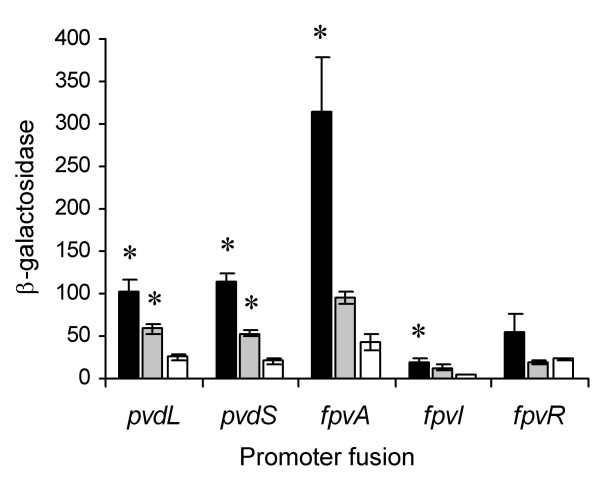
**PVD gene activities in response to iron**. The activities of *pvdL*, *pvdS*, *fpvA*, *fpvI *and *fpvR *in response to iron were determined from '*lacZ *reporter fusion strains, TR107.2.1, TR107.1.1, TR135.1.1, TR107.4.1, and TR107.5.1, respectively. β-galactosidase activity (fmol 4 MU/min/cell) was assayed for cells growing in CAA media supplemented with 100 μM 2,2'-dipyridyl (black bars), 450 nM FeSO_4 _(hatched bars), and 450 μM FeSO_4 _(white bars). Data are the means ± the standard errors of four independent experiments for each strain. Asterisks indicate that the promoter activity in the given iron treatment (100 μM 2,2'-dipyridyl or 450 nM FeSO_4_) was significantly different to the background activity in 450 μM FeSO_4 _at the P = 0.05 level of significance using Dunnett's method.

To ascertain whether the SBW25 PVD gene homologues identified by *in silico *analyses are responsive to iron, a selection of genes were assessed using chromosomally integrated '*lacZ *transcriptional fusions. Genes were chosen that represented key regulatory and structural components in PVD mediated iron-uptake, and included the ECF sigma factor encoding genes *pvdS *and *fpvI*, the anti-sigma factor encoding gene *fpvR*, and structural genes involved in the biosynthesis and uptake of PVD, *pvdL *and *fpvA*, respectively (fusion strains TR107.1.1, TR107.4.1, TR107.5.1, TR107.2.1, and TR135.1.1 respectively; Table 1). The β-galactosidase activity of each fusion strain was assessed under high iron (450 μM FeSO_4_), moderate iron (450 nM FeSO_4_), and low iron (100 μM 2,2'-dipyridyl) conditions. All gene fusions showed decreasing levels of activity as iron concentration was increased (Figure 2), which is consistent with genes that are directly or indirectly under the control of the Fur repressor. The most active gene tested was *fpvA*, the activity of which was almost 3-fold greater than the next most active gene, *pvdS*.

**Table 1:** Bacterial strains and plasmids used in this study.

Strain/plasmid	Relevant genotype and/or other characteristics	Reference
***P. fluorescens***		
SBW25	Wild-type	[17]
PBR840	SBW25Δ*pvdL *or Δ*Pflu4387*	This study
15F3	PBR840 with mini-Tn *phoA3 *disruption of secondary siderophore biosynthesis genes, Gm^R^	S. Matthijs, unpublished
TR107.1.1^a^	SBW25 carrying the *pvdS*-'*lacZ *fusion, Sm^R^	This study
TR107.2.1^a^	SBW25 carrying the *pvdL-'lacZ *fusion, Sm^R^	This study
TR107.4.1^a^	SBW25 carrying the *fpvI*::'*lacZ *fusion, Sm^R^	This study
TR107.5.1^a^	SBW25 carrying the *fpvR*::*'lacZ *fusion, Sm^R^	This study
TR135.1.1^a^	SBW25 carrying the *fpvA*::*'lacZ *fusion, Sm^R^	This study
TR137.2.1	TR107.2.1 with pTr130.1, Gm^R^	This study
TR137.3.1	TR107.2.1 with pTr130.2, Gm^R^	This study
TR137.4.1	TR107.2.1 with pTr130.3, Gm^R^	This study
TR156.1.1	TR107.2.1 with pBroadgate-D, Gm^R^	This study
TR138.2.1	TR135.1.1 with pTr130.1, Gm^R^	This study
TR138.3.1	TR135.1.1 with pTr130.2, Gm^R^	This study
TR138.4.1	TR135.1.1 with pTr130.3, Gm^R^	This study
TR156.3.1	TR135.1.1 with pBroadgate-D	This study
TR168.1.1	TR107.5.1 with pTr130.1, Gm^R^	This study
TR168.2.1	TR107.5.1 with pTr130.2, Gm^R^	This study
TR168.3.1	TR107.5.1 with pTr130.3, Gm^R^	This study
TR168.4.1	TR107.5.1 with pBroadgate-D	This study
**Plasmids**		
pUIC3	Integration vector with *'lacZ*, Tc^R^	[20]
pHP45Ω	Source of the omega cassette, Sm^R^	[51]
pIVET-Sm	pUIC3 with *tet *gene replaced by Sm^r^/Sp^r ^Ω fragment from pHP45Ω, Sm^R^	This study
pUIC3–40	pUIC3 containing *pvdL *deletion fragment	This study
pCR2.1	PCR product cloning vector, Km^R^	Invitrogen
pRK2013	helper plasmid, Km^R^, Tra^+^	[52]
pTR100.1.1	pIVET-Sm carrying the *pvdS*-'*lacZ *fusion	This study
pTR100.2.1	pIVET-Sm carrying the *pvdL*-'*lacZ *fusion	This study
pTR100.4.1	pIVET-Sm carrying the *fpvI-'lacZ *fusion	This study
pTR100.5.1	pIVET-Sm carrying the *fpvR-'lacZ *fusion	This study
pTR128.1.1	pIVET-Sm carrying the *fpvA-'lacZ *fusion	This study
pBroadgate	Gateway expression vector with P_lac _promoter, Gm^R^	Thwaites and Mansfield
pBroadgate-D	pBroadgate with *ccdB *gene replaced with the linker fragment, Gm^R^;	[53]
pTR130.1	pBroadgate::*pvdS*, Gm^R^	This study
pTR130.2	pBroadgate::*fpvI*, Gm^R^	This study
pTR130.3	pBroadgate::*fpvR*, Gm^R^	This study

^a ^The *'lacZ *fusion reporter was integrated into the PVD locus by insertion-duplication thus the gene function was not affected.

**Figure 3:**
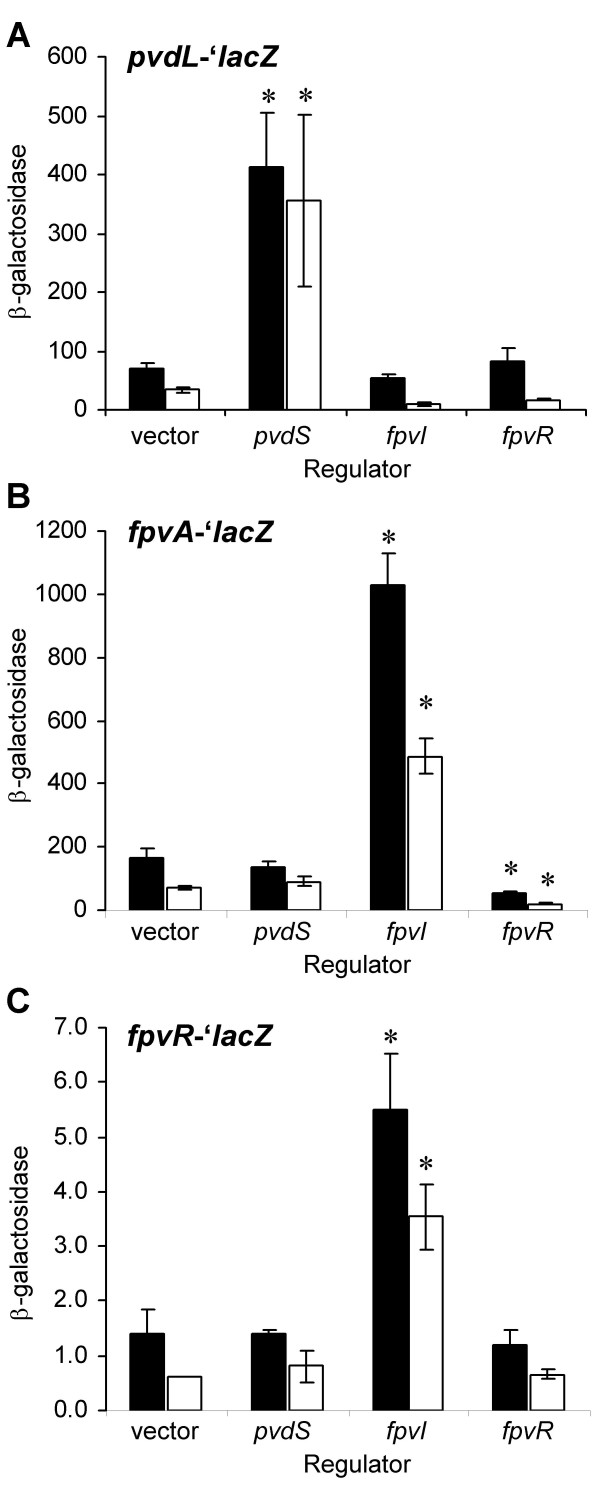
**PVD gene activities in response to constitutively-expressed putative regulators**. The activities of *pvdL-'lacZ *(**A**), *fpvA-'lacZ *(**B**), and *fpvR-'lacZ *(**C**) (fusion strains: TR107.2.1, TR135.1.1, and TR107.5.1, respectively), in response to constitutively-expressed regulator genes *pvdS*, *fpvI*, and *fpvR *(in expression constructs pTR130.1, pTR130.2 and pTR130.3, respectively). β-galactosidase activity (fmol 4 MU/min/cell) was assayed for cells growing in CAA media supplemented with 100 μM 2,2'-dipyridyl (black bars), or 450 μM FeSO_4 _(white bars). Data are the means ± the standard errors of four independent experiments. Asterisks indicate that under the given iron regime, the reporter strain activity with expression construct significantly differs from the background activity of the reporter strain containing the pBroadgate-D (vector) using Dunnett's method at the P = 0.05 level of significance.

To determine whether SBW25 possesses a branched Fpv signaling pathway analogous to that in PAO1 [5], the effects of the two constitutively expressed ECF sigma factors (PvdS, FpvI) and the anti-sigma factor (FpvR) on downstream structural genes (*pvdL *and *fpvA*) were assessed. Expression constructs (pTR130.1, pTR130.2 and pTR130.3 for *pvdS*, *fpvI *and *fpvR *respectively) were created in the vector pBroadgate which resulted in the cloned genes coming under the control of the constitutive P_lac _promoter. The expression constructs, and corresponding control vector, pBroadgate-D, were introduced into the *pvdL-'lacZ *(TR107.2.1) and *fpvA-'lacZ *(TR135.1.1) fusion strains, and their activities were monitored in β-galactosidase assays under high and low iron regimes in CAA medium. Expression of *pvdL *and *fpvA *was significantly increased as a consequence of constitutive expression of *pvdS *and *fpvI*, respectively (Figure 3A and 3B), consistent with the notion that PvdS and FpvI are alternative ECF sigma factors that direct regulation of each of these genes. In both cases, an increase in expression was observed even in the presence of iron, consistent with Fur-independent regulation of the cloned regulators. Furthermore, expression of *fpvR *caused a significant decrease in the level of *fpvA *expression, consistent with FpvR being an anti-sigma factor in the Fpv signaling pathway model [5]. For *pvdL*, repression by FpvR was observed under high iron conditions, though not to a statistically significant level. Thus to verify the presence of the Fpv branched signaling pathway in SBW25, PVD induction of the pathway was demonstrated by providing purified SBW25 PVD to the *pvdL-'lacZ *and *fpvA-'lacZ *fusion strains. Here, the exogenous PVD stimulated the signaling pathway, resulting in significant up-regulation of both *pvdL *(1.9-fold) and *fpvA *(3.0-fold; both results significant at P = 0.05 using a one-tail t-test). Taken together, these data are consistent with SBW25 possessing a branched Fpv signaling pathway that is activated by PVD.

### Unique regulation of *fpvR *in SBW25

Next we tested the hypothesis that *fpvR *is regulated by PvdS rather than Fur, which is the direct regulator of anti-sigma factors in many other bacterial iron-acquisition systems [27]. The regulatory gene constitutive expression constructs (pTR130.1, pTR130.2 and pTR130.3 for *pvdS*, *fpvI *and *fpvR *respectively) were introduced into the *fpvR*-'*lacZ *fusion strain (TR107.5.1). β-galactosidase activities of the resulting strains were determined under high (450 μM FeSO_4_) and low (100 μM 2,2'-dipyridyl) iron regimes. Contrary to expectation, the results presented in Figure 3C showed that expression of FpvI, and not PvdS, resulted in the up-regulation of *fpvR*, even in the presence of iron. It is unknown whether FpvI directly regulates *fpvR*, or if an intermediate regulatory component is involved. However, a conserved sequence motif, 5'-TAATGAGAA-3', was identified 56 nt upstream of *fpvA *and 128 nt upstream of *fpvR*, which may potentially serve as an FpvI binding site.

Genomic comparisons suggest that the non-adjacent arrangement of *fpvI *and *fpvR *in SBW25 may not be unique. Homologues of *fpvR *were not reported in *P. putida *KT2440 and *P. fluorescens *Pf0–1, and *fpvR *and *fpvI *were not identified in *P. syringae *pv. tomato DC3000 [23], however, this is likely to reflect a lack of strong sequence homology to the characterized counterparts in PAO1. Indeed, in SBW25, the amino acid sequence identity between FpvR and its PAO1 homologue was the lowest among all genes examined (48.7%; Additional file 1). A search for *fpvR *genes in other sequenced *P. fluorescens *strains revealed putative homologues in Pf0–1 and Pf-5 (48.1% and 48.4% amino acid sequence identity to PAO1 FpvR, respectively), however neither gene was adjacent to its cognate *fpvI *homologue, nor possessed a recognizable Fur-box motif in the upstream sequence. A putative IS-box was observed 44 bp upstream of the Pf0–1 homologue, with 7 out of 8 bases matching the IS-box consensus. However, an IS-box was not observed for the Pf-5 homologue, nor was the potential FpvI-binding site sequence identified upstream of SBW25 *fpvA *and *fpvR*. The presence of key genes encoding components of the Fpv branched signaling pathway (*fpvA*, *fpvR*, *pvdS *and *fpvI*) in the Pf0–1 and Pf-5 genomes suggests that this pathway may be conserved in these *P. fluorescens *strains – a hypothesis that can be tested in future work.

### Deletion analysis of *pvdL*

**Figure 4:**
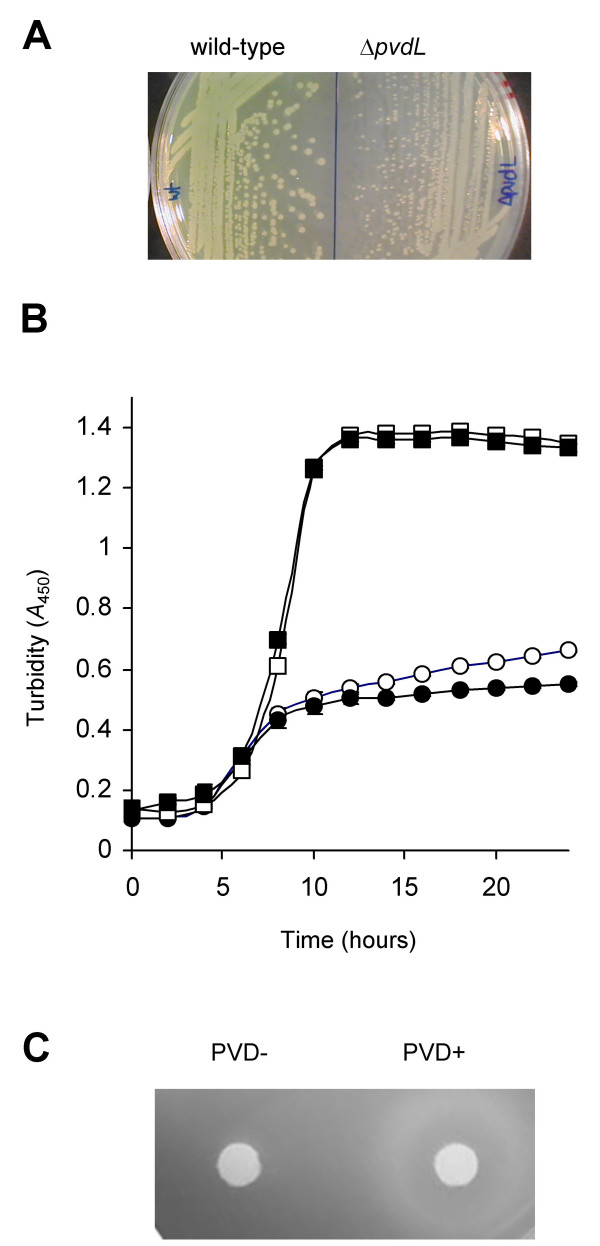
**Growth phenotype of mutant PBR840 (Δ*pvdL*) in iron-limited media**. **A: **Growth of SBW25 and PBR840 on LB agar for two days, after which the mutant produced no visible PVD and colonies were smaller than wild-type. **B: **Growth in CAA broth (SBW25, clear circle; PBR840, filled circle) and in CAA broth supplemented with 450 μM FeCl_3 _(SBW25, clear square; PBR840, filled square). Results are means and standard errors from at least five independent cultures (error bars are contained within the symbols). Data were collected at 5 minute intervals, but 2 hourly time points are shown for clarity. Data from each medium were analyzed separately using a multivariate model (MANOVA) with repeated measures (2 h intervals): no significant difference was detected between genotypes grown in CAA supplemented with FeCl_3 _[F_1,8 _= 0.03, *P *= 0.862], however, a significant genotype effect was evident in iron deficient medium [F_1,8 _= 37.22, *P *< 0.001]. An analysis at each time point revealed a highly significant difference between genotypes from (and including) the 14 h time point [F_1,8 _= 28.02, *P *< 0.0001]. **C**: PBR840 cells were plated on CAA media with EDDHA and overlaid with filter paper disks impregnated with sterile water (left) and 50 nmol purified SBW25 PVD (right). PBR840 was not able to grow on the strongly iron-chelated media, growth was restored when supplemented with PVD.

To investigate the biological roles of the PVD genes predicted from *in silico *analysis, an SBW25 derivative strain containing a deletion of *pvdL *was constructed (PBR840, Δ*pvdL*). In SBW25, *pvdL *(Pflu4387) is predicted to encode a 4297 amino acid NRPS involved in PVD chromophore biosynthesis. PBR840 displayed a PVD negative phenotype when grown on LB agar: wild-type SBW25 produced a typical yellow-green siderophore, whereas PBR840 did not (Figure 4A). It was also observed that colonies of PBR840 were notably smaller than those of SBW25 (Figure 4A). It is unknown whether this is solely due to impairment in iron uptake, or whether an iron-independent factor negatively affects PBR840 growth.

The growth kinetics of PBR840 were determined in iron-limited CAA broth and the same medium supplemented with 450 μM FeCl_3 _in microtitre plate cultures. Results are shown in Figure 4B. A repeated measures MANOVA (see figure caption for details) showed that the optical density of PBR840 cells was significantly reduced compared to wild-type in iron deplete medium from 14 h [F_1,8 _= 28.02, *P *< 0.0001]. No significant reduction was detected during exponential growth, for example, at 4, 6 and 8 h, *P *values were 0.849, 0.333 and 0.398, respectively. No significant difference in growth was observed between wild-type and PBR840 in iron replete medium [F_1,8 _= 0.03, *P *= 0.862].

Growth analysis of PBR840 took place initially in 200 μl microtitre plate cultures, which although incubated with periodic shaking, could have become oxygen limited thus masking the effect of the *pvdL *mutation during exponential growth. To test this hypothesis, growth of PBR840 and SBW25 was further investigated using strongly aerated cultures grown in CAA broth, where iron is more likely to be present in the ferric form. The growth dynamics (data not shown) were comparable to those observed in the static cultures, with no statistical difference in cell density being detected during exponential growth, however differences in final yield were evident at 24 hours (the mean A450 reading and standard error for PBR840 was 0.9239 ± 0.014 (n = 4), and for SBW25 was 1.3510 ± 0.016 (n = 4)). Furthermore, PBR840 was incapable of growing in CAA medium supplemented with the strong iron-chelator EDDHA (ethylenediaminedihydroxyphenylacetic acid, 0.5 mg/ml), whereas growth was restored upon provision of purified SBW25 PVD (Figure 4C). Taken together, the data confirmed the predicted role of *pvdL *(Pflu4387) in PVD production.

### Structural analysis of SBW25 PVD

**Figure 5:**
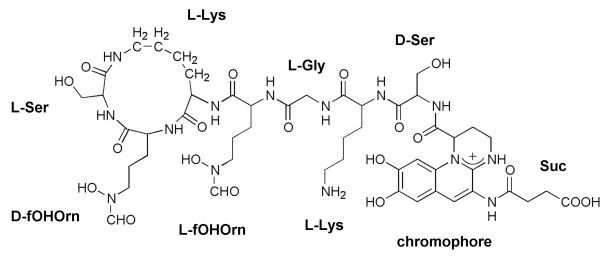
**Chemical structure of *P. fluorescens *SBW25 PVD**. Structure of SBW25 PVD with succinic acid (amide) side chain (Suc) amidically connected to the chromophore shown. The side chain may also comprise ketoglutaric acid. fOHOrn, *N*5-formyl-*N*5-hydroxyornithine.

The chemical structure of SBW25 PVD was determined by mass spectral analysis using standard methods as previously described [28]. Comparison of the mass spectra to published data [29-32] revealed that SBW25 PVD comprises the characteristic PVD quinoline chromophore [33] linked to a partly cyclic seven residue peptide backbone, identical to that of *P. fluorescens *ATCC 13525 [34]: Ser-Lys-Gly-fOHOrn-c(Lys-fOHOrn-Ser) (fOHOrn = *N*^5^-formyl-*N*^5^-hydroxyornithine and underlined residues are in the D configuration) (Figure 5). The side chain that is connected amidically to the NH_2_-group of the chromophore varied as succinic acid or ketoglutaric acid. The peptide backbone sequence differs in length and composition to that of PAO1 PVD (Ser-Arg-Ser-fOHOrn-c(Lys-fOHOrn-Thr-Thr) [35]). *In silico *analyses [36,37] of the protein sequences of the putative PVD NRPSs: PvdI, Pflu2544, Pflu2552, Pflu2553 and PvdL, were performed to predict their amino acid substrates (results are shown in Additional file 2). Comparison of the substrate predictions with the actual PVD structure suggested that only PvdI and Pflu2544 are involved in the biosynthesis of the SBW25 PVD backbone, with predictions for three of the amino acid residues being correct (Ser, Gly and Ser). Dhb/sal-like residues were consistently predicted for both occurrences of fOHOrn for PvdI and Pflu2544 (see Additional file 2). It is unclear why the algorithm has made this prediction, as fOHOrn does not share similar physico-chemical properties with dhb and sal, however, similar analyses of the PAO1 PVD NRPSs have also consistently predicted dhb/sal-like specificities for the fOHOrn binding modules (Additional file 2). The roles of Pflu2552 and Pflu2553 are presently unknown, however the presence of an IS-box-like motif preceding these genes, and their proximity to other PVD gene homologues (Figure 1) suggests that they have, or have had, a role in PVD-mediated iron-uptake processes. As the SBW25 PVD chromophore structure is identical to that of PAO1 PVD [33], it was not surprising that the *in silico *substrate predictions for SBW25 PvdL were the same as those for PAO1 PvdL (Additional file 2), although only one of the three residues was predicted correctly (glu) in each case.

### Utilization of exogenous PVDs by *P. fluorescens *SBW25

The identification of two SBW25 plant-induced genes with homology to siderophore receptor genes [19,20] prompted us to examine the ability of SBW25 to uptake and utilize exogenous siderophores. Initially, a genome-wide search for putative TonB-dependent siderophore receptor genes was undertaken, which revealed 24 putative siderophore receptor genes dispersed throughout the genome (listed in Additional file 3). Nine of these were immediately adjacent to predicted sigma factor/anti-sigma factor gene pairs (Additional file 3), that are presumably involved in their regulation. The number of putative receptor genes is similar to that (26) for *P. fluorescens *Pf0–1 [3], and include *fpvA *(Pflu2545), and Pflu5798 and Pflu6132 which were both previously shown to be induced *in planta *[19]. These results strongly support the notion that SBW25 has the capability to utilize a considerable number of exogenous iron-chelating siderophores produced by other microbes.

To test the ability of SBW25 to utilize exogenous PVDs, PVDs purified from 25 different *Pseudomonas *isolates (Table 2), each with a different siderotype profile (P. Cornelis, personal communication), were supplied to 15F3 (a secondary siderophore-deficient mutant strain of PBR840) that had been overlaid as a dilute suspension onto CAA medium chelated with EDDHA. The mutant was unable to grow when water was provided as a negative control. However, growth was restored by provision of PVDs purified from 19 different isolates (Table 2). These include the three types (I, II and III) of PVD from *P. aeruginosa*. Thus, it is apparent that SBW25 is able to utilize a range of exogenous PVDs produced by other pseudomonads.

**Table 2:** Utilization of exogenous *Pseudomonas *PVDs

Strain PVD isolated from	15F3 growth stimulation	Reference
*P. aeruginosa *PAO1^a^	+	[40, 54]
*P. aeruginosa *7NSK2^a^	+	[55]
*P. aeruginosa *59.20^a^	+	[39]
*P. agarici *LMG 2112	+	BCCM/LMG^b^
*P. asplenii *LMG 5147	-	BCCM/LMG
*P. fluorescens *LMG 14562	+	BCCM/LMG
*P. fluorescens *17400::*qbsF*^c^	-	[56]
*P. fluorescens *5	+	A. Sarniguet, INRA, France
*P. fluorescens *C7R12	+	P. Lemanceau, INRA/Université de Bourgogne, France
*P. fluorescens *SBW25	+	[17]
*P. libanensis *LMG 21606	-	BCCM/LMG
*P. salomonii *LMG 22120	+	BCCM/LMG
*P. syringae *LMG 1247	+	BCCM/LMG
*P. vancouverensis *LMG 20222	-	BCCM/LMG
*Pseudomonas *sp. W2Ap9	+	S. Matthijs, VUB, Brussels
*Pseudomonas *sp. W2Aug1	+	S. Matthijs, VUB, Brussels
*Pseudomonas *sp. W2Aug7	+	S. Matthijs, VUB, Brussels
*Pseudomonas *sp. W2Aug36	+	S. Matthijs, VUB, Brussels
*Pseudomonas *sp. W2Dec18	+	S. Matthijs, VUB, Brussels
*Pseudomonas *sp. W2Dec29	+	S. Matthijs, VUB, Brussels
*Pseudomonas *sp. W2Dec33	-	S. Matthijs, VUB, Brussels
*Pseudomonas *sp. W15Feb31B	+	S. Matthijs, VUB, Brussels
*Pseudomonas *sp. W2Jun14	-	S. Matthijs, VUB, Brussels
*Pseudomonas *sp. W15Ap2	+	S. Matthijs, VUB, Brussels
*Pseudomonas *sp. W15Aug 1	+	S. Matthijs, VUB, Brussels
*Pseudomonas *sp. W15Oct32	+	S. Matthijs, VUB, Brussels

^a ^*P. aeruginosa *strains PAO1, 7NSK2 and 59.20 produce types I, II and III PVDs, respectively.

^b ^BCCM/LMG: Belgian co-ordinated collections of micro-organisms bacteria collection

^c ^Secondary siderophore (thioquinolobactin) production is inactivated in this strain.

## Conclusion

The genomic era has revolutionized the scientific method of comparative biology, where the general applicability of biological traits and systems that have often been evaluated in few model organisms may now be readily assessed across taxa. Although PVDs are common siderophores of *Pseudomonas *spp., the regulation of the genes involved in the biosynthesis and uptake of PVD has been elucidated almost exclusively in *P. aeruginosa *PAO1. The availability of the *P. fluorescens *SBW25 genome sequence has revealed that PVD genes are considerably more widespread throughout the SBW25 genome than in other sequenced pseudomonads, and has enabled a comparative analysis of PVD regulation in SBW25 and PAO1 to be undertaken. The PVD regulatory model established for PAO1 involves a unique branched signaling pathway [5]. Prior to this study it was not known whether this model applies to other *Pseudomonas *isolates. Using chromosomally-integrated gene reporter fusions and gene expression constructs, we found that the PAO1 branched signaling model is present in SBW25. In addition, we reveal a significant difference between PAO1 and SBW25 in terms of the regulation of *fpvR*. In SBW25, over-expression of *fpvI *results in constitutive expression of *fpvR*, suggesting that *fpvR *is positively regulated by FpvI, rather than directly by Fur, as is the case of *fpvR *of PAO1 [5]. The structure of SBW25 PVD was elucidated and it was found that the peptide backbone is identical to that of *P. fluorescens *ATCC 13525, comprising a partly cyclic peptide of seven residues. The PVD structure implicated the involvement of two NRPSs in the synthesis of the PVD peptide backbone. The SBW25 genome harbors 24 putative TonB-dependent siderophore receptors, two of which are previously shown to be plant-inducible [19]. Exogenous PVD uptake assays demonstrate that SBW25 is able to utilize a variety of structurally distinct siderophores produced by other *Pseudomonas *isolates. These data lay the foundation for further detailed functional analysis of these putative siderophore receptors in term of regulation and transporter specificity.

## Methods

### Bacterial strains, plasmids, and growth conditions

*Escherichia coli *DH5αλ*pir *[38] was used as a recipient strain for gene cloning and then a donor for conjugative transfer into *Pseudomonas *cells. *Pseudomonas *strains and plasmids used in this study are listed in Table 1. *E. coli *cultures were grown at 37°C in Luria-Bertani (LB) medium [38] and *P. fluorescens *cultures were grown at 28°C in LB or casamino acids medium (CAA) [39]. Antibiotics were used at the following final concentrations (μg/ml): ampicillin (Ap) 100, chloramphenicol (Cm) 25, gentamycin (Gm) 20, kanamycin (Km) 50, streptomycin (Sm) 100 and tetracycline (Tc) 10. 5-Bromo-4-chloro-3-indolyl β-D-galactopyranoside (X-gal; Melford Laboratories, Chelsworth, UK) was used at a final concentration of 40 μg/ml.

To monitor PVD gene activities, reporter strains were grown in the presence of 450 nM – 450 μM FeSO_4 _for iron supplemented media. To chelate iron, 100 μM 2, 2'-dipyridyl (Sigma, St Louis MO, USA), or 0.5 mg/ml ethylenediaminedihydroxyphenylacetic acid (EDDHA) was used. Purified PVD was supplied at 50 μM.

Growth curves of bacterial strains were obtained using a VersaMax microtiter plate reader with SOFTmax PRO software (Molecular Devices, Sunnyvale CA, USA). Inoculum was prepared by culturing strains stored at -80°C in LB broth overnight, then sub-culturing into CAA broth with growth overnight. For growth experiments, cells from the CAA culture were washed once with sterile water and 2 μl was used inoculate 200 μl CAA or CAA supplemented with 450 μM FeCl_3 _in wells of the 96-well microtiter plate. Plates were incubated at 28°C and absorbance readings at 450 nm were taken every five minutes after brief shaking. Growth curves were also obtained from highly aerated cultures grown in 250 ml flasks containing 20 ml CAA broth, and inoculated to an initial OD450 of ~0.15. Cultures were grown at 28°C with shaking at 200 rpm and absorbance readings at 450 nm were taken over a 24 hour period.

### *In silico *analyses

Putative homologues of PVD-related genes in *P. fluorescens *SBW25 were identified by interrogating the un-annotated SBW25 genome [22] with *P. aeruginosa *PAO1 PVD gene sequences [23,40] using nucleotide-nucleotide Basic Local Alignment Search Tool (BLASTN). Complete SBW25 open reading frames (ORFs) were identified from matching sequence hits, and these were used to re-query the PAO1 genome *via *translated query vs. protein sequence BLAST (tBLASTX) algorithms. Sequences upstream of putative homologues were examined by eye for the PAO1 Fur-box consensus sequence, 5'-GATAATGATAATCATTATC-3' [11], and iron starvation (IS) boxes, 5'-TAAAT-N_16_-CGT-3' [12,41]. The best matches to Fur-box motifs were confirmed using MotifScanner [42], in conjunction with a Fur-box position weight matrix (accession MX000013) from the Prodoric website [43,44]. Genes were mapped to the genome using Artemis software [45], and the percentage identity and similarity at the amino acid sequence level between PAO1 and SBW25 homologues was determined by the Needleman-Wunsch global alignment algorithm implemented in EMBOSS needle [46]. Common DNA sequence motifs were identified by MEME version 3.0 [37]. *In silico *PVD peptide backbone structural predictions were conducted using the methods of Rausch *et al.* and Stachelhaus *et al.*[36,37] implemented in NRPSpredictor [47]. Putative siderophore receptor genes were identified by hits to protein family (Pfam) accessions PF00593 (TonB-dependent receptor) and PF07715 (TonB-dependent receptor plug domain) [22], in combination with BLAST homology to putative siderophore receptor genes. Putative sigma factor and anti-sigma factor genes adjacent to receptor genes were identified by hits to Pfam accessions PF04542 (Sigma-70 region) and PF04773 (FecR protein), respectively.

### Gene reporter and expression constructs

DNA manipulation was performed by standard techniques [38]. Promoter reporter fusion strains were generated by cloning the promoter region in the front of a promoterless '*lacZ *carried in the cloning vector pIVET-Sm, a Sm^R ^derivative of pIVETD [19]. Promoter regions were created by PCR amplifying regions upstream of, and including the 5'-portion of PVD genes using primers listed in Table 3. To facilitate directional cloning, *Bgl*II and *Xho*I sites were incorporated into the 5'-ends of the sense and anti-sense strand primers, respectively. PCR products were digested with *Bgl*II and *Xho*I, and ligated to *Bgl*II-*Xho*I digested pIVETD-Sm, creating fusions to promoterless *'lacZ*. Reporter strains were created by transforming SBW25 with the promoter fusion constructs *via *electroporation or conjugation. Transformants were selected based on streptomycin resistance and PCR screened using a primer upstream of the cloned promoter fragment and a '*lacZ *primer to confirm the construct had integrated into the genome by single-crossover homologous recombination within the cloned PVD promoter fragment region.

**Table 3:** Oligonucleotide primers used in this study.

Primer	Sequence (5'-3')^a^	Application
CM-030	GGGAGATCTAGGTCCACATTGGATTGCAGG	*pvdS *promoter
CM-038	CCGCTCGAGGGTGAAGCGCCGTGAATAACCACA	*pvdS *promoter
CM-036	GGAAGATCTCCTGTGGATACTTGTTCCGTCA	*pvdL *promoter
CM-037	CCGCTCGAGACCAAAGAACGCCGCGACGTAATC	*pvdL *promoter
CM-050	GGAAGATCTCTGCTGGGCGTTGAGGGTCTGG	*fpvI *promoter
CM-051	CCGCTCGAGCGGGAAATGCACAGGCGTTCGGCA	*fpvI *promoter
CM-052	GGAAGATCTCGTCTGCAGCGTCTGGTCCTGGAG	*fpvR *promoter
CM-053	CCGCTCGAGGTCCTTGTAGTTGCTGTAGACCAG	*fpvR *promoter
CM-116	GGGAGATCTGAAGTAGGAACGTGTCACTAGTGG	*fpvA *promoter
CM-117	GGGCTCGAGGTCGAAGTGCCTGCCAGCAAG	*fpvA *promoter
CM-110	**CACC**GTGTAGGAGGCCTCAGCAAGG	*pvdS *ORF
CM-111	ACATAAGGCGCACATTTCATCTGC	*pvdS *ORF
CM-112	**CACC**ACTCAGGGACCTAATGCCGC	*fpvI *ORF
CM-113	CATGTGTTGATAGGAAACGAGGAC	*fpvI *ORF
CM-114	**CACC**CGTGAAGTTAGAATGTGGG	*fpvR *ORF
CM-115	TGGGCGGTTCAGGGCATG	*fpvR *ORF
pvdL1	GAAGATCTTCGAACTTCCCCAAACGCT	*pvdL *deletion
pvdL2	*CAGCATGCGGATCCGTTGACGGA*ACCCGCAGCGCGGGGATGCC	*pvdL *deletion
pvdL3	*TCCGTCAACGGATCCGCATGCTG*TGGCGCACCAATGGCTGCT	*pvdL *deletion
pvdL4	GAAGATCTCCAACTCCGCCATCAAATCA	*pvdL *deletion

^a ^Underlined sequences denote *Bgl*II or *Xho*I restriction sites to facilitate cloning; sequences in bold type are tag for directional topoisomerase-mediated cloning, and sequences in italics denote random complementary sequences to facilitate SOE-PCR [50].

Gene expression constructs for *pvdS*, *fpvI *and *fpvR *were created using the Gateway cloning system (Invitrogen). Fragments containing the ribosome-binding site and entire ORF of *pvdS*, *fpvI *and *fpvR *were PCR-amplified using primers in Table 3. Fragments were directionally cloned into pENTR/D-TOPO (Invitrogen) following the manufacturers instructions. The sequences of the cloned fragments were verified, and LR recombination reactions using Gateway LR Clonase enzyme mix (Invitrogen) were performed to transfer the cloned fragment to the broad host range destination expression vector, pBroadgate (gift from R. Thwaites and J. Mansfield). pBroadgate is a derivative of pBBR1MCS-5 [48] that has been modified as a Gateway destination vector by incorporation of an *att*R cassette, containing a Cm resistance determinant and a lethal *ccd*B gene (Invitrogen). *P. fluorescens *reporter strains were transformed with expression and pBroadgate-D control constructs by electroporation.

### β-galactosidase assays

β-galactosidase activities of *'lacZ *reporter fusion strains were determined from cultures grown to mid- to late log phase in CAA media, supplemented with FeSO_4 _or 2,2'-dipyridyl as required. Assays were based on the hydrolysis of 4-methylumbelliferyl-β-D-galactoside to yield the fluorescent product, 7-hydroxy-4-methylcoumarin (4MU) as described [49]. 4MU was detected at 460 nm after excitation at 365 nm using a Hoefer DyNA Quant 200 fluorometer (Pharmacia Biotech, San Francisco CA, USA), or FLUOstar (BMG Labtech, Offenburg, Germany).

### Construction of PBR840 (Δ*pvdL*)

An ORF Pflu4387 (*pvdL*) knockout mutant was constructed by deleting a 10.9 kb internal portion of the 12.9 kb *pvdL *gene. This was achieved by a combination of gene splicing by overlap extension (SOE) PCR [50] and a two-step allelic exchange strategy [20]. Briefly, two 1 kb fragments from each of the 5'- and 3'-end of *pvdL *were PCR-amplified from SBW25 genomic DNA using the primer pairs pvdL1 and pvdL2, and pvdL3 and pvdL4, respectively (Table 3). The two fragments were ligated in a third PCR using primers pvdL1 and pvdL4 *via *the complementary residues incorporated at the 5'-ends of primers pvdL2 and pvdL3 (Table 3). The resulting 2 kb *pvdL *deletion fragment was cloned into pCR8/GW/TOPO (Invitrogen). After the sequence of the cloned fragment was verified, the fragment was retrieved by *Bgl*II digestion and cloned into the integration vector pUIC3 to create pUIC3–40.

To generate a chromosomal *pvdL *deletion, pUIC3–40 was mobilized into *P. fluorescens *SBW25 by conjugation with the help of pRK2013 (tra^+^). Chromosomal integration of pUIC3–40 by a single homologous recombination event was selected by plating transconjugants on LB agar containing Tc and X-gal. Blue, Tc-resistant colonies contained both intact and deleted versions of *pvdL*, and were grown in LB broth without selection to allow the second homologous recombination event to take place. After two consecutive overnight subcultures, cells were plated on LB agar containing X-gal. White colonies were screened for Tc sensitivity, and the *pvdL *deletion was confirmed by PCR analysis.

### PVD structure determination

To determine the structure of SBW25 PVD, mass spectral data were obtained with a MAT 900 ST instrument providing an EB-QIT (quadrupole ion trap) geometry and equipped with an ESI II ion source (Finnigan MAT, Bremen, Germany); spray voltage 3.4–3.6 kV, capillary temperature 230°C. Source conditions were set to minimize fragmentation, resolution ca. 5000 (10% valley). Trifluoroacetic acid was added to the sample dissolved in water (100:0.2, v/v). Fragmentation induced by low energy collision activation was effected in the octapole unit, located in front of the QIT and in the QIT itself (~2·10-3 Pa He as bath gas diffusing in the collision octapole).

### PVD purification and exogenous siderophore uptake by SBW25

PVD was purified from bacterial cultures grown for 48 h in 1 l of CAA at 28°C. For *P. aeruginosa*, the strains were grown at 37°C. The cell-free supernatant was passed on a C-18 column (5 × 2.5 cm) conditioned with methanol and rinsed with sterile H_2_O. PVD was eluted with 80% methanol. Subsequently the methanol was evaporated and the PVD lyophilized. Purified PVD was quantified spectrophotometrically [39].

To estimate the diversity of exogenous PVDs that may be utilized by SBW25, PVDs were purified from a panel of 25 *Pseudomonas *isolates and strains, representing a high diversity of species that had previously been shown to each produce a unique PVD (each having a unique siderotype) as shown by isoelectric focusing (S. Matthijs, unpublished). These isolates included *Pseudomonas *type strains, and environmental isolates collected from the Woluwe river, Brussels, Belgium (Table 2). CAA agar plates containing 0.5 mg/ml EDDHA were overlaid with 5 × 10^6 ^cells of strain 15F3, a siderophore-deficient derivative of PBR840 that contains transposon mini-Tn *phoA3 *disruption of biosynthetic genes for a secondary siderophore (S. Matthijs, unpublished). Filter-paper disks impregnated with 5 μl of 8 mM purified siderophore were placed on the agar [39]. Plates were incubated at 28°C and scored for the presence of detectable growth after one day.

## List of abbreviations used

CAA, casamino acids medium; EDDHA, ethylenediaminedihydroxyphenylacetic acid; IVET, *in vivo *expression technology; LB, Luria-Bertani; NRPS, non-ribosomal peptide synthetase; PCR, polymerase chain reaction; PVD, pyoverdine; QIT, quadrupole ion trap; SOE, splicing by overlap extension; X-gal, 5-bromo-4-chloro-3-indolyl β-D-galactopyranoside; 4MU, 7-hydroxy-4-methylcoumarin.

## Authors' contributions

CDM conducted the bioinformatic and genetic work, and drafted the manuscript. XXZ created the Δ*pvdL *mutant, assessed growth characteristics, and contributed to the manuscript. SM performed the assay for the heterologous usage of PVDs. MS and HB determined the structure of SBW25 PVD. PBR assisted with experimental design and critically reviewed the manuscript. All authors have read and approved the final manuscript.

## Supplementary Material

Additional file 1 List of *P. fluorescens *SBW25 genes with predicted function in PVD biosynthesis and uptake in comparison with homologues in *P. aeruginosa *PAO1. The data list all putative PVD genes identified in SBW25, and show predicted amino acid identities to their PAO1 homologues. Click here for file

Additional file 2 NRPS substrate *in silico *predictions. The data show the substrates of the PVD NRPS modules predicted by specificity conferring code and transductive support vector machine methods. Click here for file

Additional file 3 List of putative siderophore receptors in *P. fluorescens *SBW25. The data list all putative siderophore receptors in the SBW25 genome, and whether these are adjacent to predicted sigma factor/anti-sigma factor gene pairs. Click here for file
