# Supplementary material for: Genomic, genetic and structural analysis of pyoverdine-mediated iron acquisition in the plant growth-promoting bacterium Pseudomonas fluorescens SBW25
Source: BMC Microbiol. 2008 Jan 14;8:7. doi: 10.1186/1471-2180-8-7 (PMC2235872; doi:10.1186/1471-2180-8-7)
Supplement: Additional file 1 — List of P. fluorescens SBW25 genes with predicted function in PVD biosynthesis and uptake in comparison with homologues in P. aeruginosa PAO1. The data list all putative PVD genes identified in SBW25, and show predicted amino acid identities to their PAO1 homologues. [file 1471-2180-8-7-S1.doc]

### Additional File 1 – List of *P. fluorescens* SBW25 genes with predicted function in PVD biosynthesis and uptake in comparison with homologues in *P. aeruginosa* PAO1

| Pflu#^a^ | Gene | Product (predicted role) | Length (bp) | Gene regulation^b^ | PA#^c^ | Amino acid sequence identity (%) |
| --- | --- | --- | --- | --- | --- | --- |
| 2041-2047 |  | Membrane proteins | 915, 900, 747, 972, 561, 330 & 537 | op. Pflu2048 | 2410-2404 | 55.3, 87.7, 72.6, 79.4, 50.0, 69.3 & 74.0 |
| 2048 |  | Membrane protein | 1203 | IS (7/8) | 2403 | 70.3 |
| 2543 | *pvdI* | NRPS | 14040 | IS (6/8) | 2402 | 52.1 |
| 2544 |  | NRPS | 11850 | op. *pvdI* |  |  |
| 2545 | *fpvA* | PVD receptor | 2346 |  | 2398 | 69.1 |
| 2546 | *pvdE* | ABC transporter | 1653 | IS (8/8) | 2397 | 77.1 |
| 2547 | *pvdF* | Transformylase | 831 | IS (8/8) | 2396 | 79.4 |
| 2548 | *pvdO* | function unknown | 861 | op. *pvdM* | 2395 | 73.1 |
| 2549 | *pvdN* | Aminotransferase | 1272 | op. *pvdM* | 2394 | 60.6 |
| 2550 | *pvdM* | Dipeptidase | 1356 | IS (8/8) | 2393 | 74.3 |
| 2551 | *pvdP* | function unknown | 1626 | IS (8/8) | 2392 | 66.5 |
| 2552 |  | NRPS | 12885 | IS (6/8) |  |  |
| 2553 |  | NRPS | 11322 | op. Pflu2552 |  |  |
| 2702 | *fpvR* | Anti-sigma factor | 996 | IS (6/8) | 2388 | 48.7 |
| 3353 | *pvdQ* | Acylase | 2286 | IS (8/8)^d^ | 2385 | 54.3 |
| 3975 | *pvdA* | L-ornithine hydroxylase | 1338 | IS (8/8) | 2386 | 74.7 |
| 3976 | *fpvI* | ECF sigma factor | 483 | Fur (12/19) | 2387 | 69.6 |
| 3977 |  | Membrane efflux | 1170 | IS (8/8) | 2389 | 72.3 |
| 3978 |  | ABC transporter | 1971 | op. Pflu3977 | 2390 | 79.7 |
| 3979 | *opmQ* | Porin | 1404 | op. Pflu3977 | 2391 | 60.4 |
| 4377 |  | MtbH | 222 | IS (7/8) | 2412 | 83.8 |
| 4378 | *pvdH* | Aminotransferase | 1395 | IS (8/8) | 2413 | 83.7 |
| 4387 | *pvdL* | NRPS | 12891 | op. *pvdG* | 2424 | 74.1 |
| 4388 | *pvdG* | Thioesterase | 753 | IS (7/8) | 2425 | 51.4 |
| 4389 | *pvdS* | ECF sigma factor | 552 | Fur (14/19) | 2426 | 84.7 |

^a^ Gene identifiers (Pflu#’s) designated by the Sanger Institute [22].

^b^ Gene regulation as predicted by the presence of IS and Fur box motifs, where the proportion of bases matching the IS and Fur box consensus sequences (5’-TAAAT-N_16_-CGT-3’ [12, 41], and 5’-GATAATGATAATCATTATC-3’ [11], respectively) are shown in brackets. In addition, genes predicted to be operonic with (op.) neighbouring genes [23] are indicated.

^c^ PVD genes from *Pseudomonas aeruginosa* PAO1 as summarized by Ravel & Cornelis [23], except *pvdJ*, *pvdD*, and PA2411, for which SBW25 homologues were not identified.

^d^ Two IS-box motifs were found upstream of *pvdQ*.
