# Supplementary material for: Genomic, genetic and structural analysis of pyoverdine-mediated iron acquisition in the plant growth-promoting bacterium Pseudomonas fluorescens SBW25
Source: BMC Microbiol. 2008 Jan 14;8:7. doi: 10.1186/1471-2180-8-7 (PMC2235872; doi:10.1186/1471-2180-8-7)
Supplement: Additional file 2 — NRPS substrate in silico predictions. The data show the substrates of the PVD NRPS modules predicted by specificity conferring code and transductive support vector machine methods. [file 1471-2180-8-7-S2.doc]

### Additional File 2 – NRPS substrate *in silico* predictions

| NRPS/Module# | Specificity-conferring code method^a^ | TSVM method (score)^b^ | SBW25 PVD sequence match^c,d^ |
| --- | --- | --- | --- |
| PvdI Pflu2543/1 | ser | ser=thr=ser-thr=dht=dhpg=dpg=hpg-like specificity (1.476) | ser |
| PvdI Pflu2543/2 | - | val=leu=ile=abu=iva-like specificity (0.943) | lys |
| PvdI Pflu2543/3 | gly | gly=ala-like specificity (1.330) | gly |
| PvdI Pflu2543/4 | - | dhb=sal-like specificity (1.178) | fOHOrn |
| Pflu2544/1 | - | asp=asn-like specificity (1.257) | lys |
| Pflu2544/2 | - | dhb=sal-like specificity (1.178) | fOHOrn |
| Pflu2544/3 | ser | ser=thr=ser-thr=dht=dhpg=dpg=hpg-like specificity (0.935) | ser |
| Pflu2552/1 | thr | thr=dht-like specificity (1.248) |  |
| Pflu2552/2 | val | gly=ala=val=leu=ile=abu=iva-like specificity (2.440) |  |
| Pflu2552/3 | leu | gly=ala=val=leu=ile=abu=iva-like specificity (1.612) |  |
| Pflu2552/4 | ser | ser-like specificity (1.502) |  |
| Pflu2553/1 | leu | gly=ala=val=leu=ile=abu=iva-like specificity (1.612) |  |
| Pflu2553/2 | ser | ser-like specificity (1.541) |  |
| Pflu2553/3 | ile | gly=ala=val=leu=ile=abu=iva-like specificity (1.387) |  |
| PvdL Pflu4387/1 | glu | asp=asn=glu=gln=aad-like specificity (1.032) | glu |
| PvdL Pflu4387/2 | lys | dhb=sal-like specificity (1.380) | tyr |
| PvdL Pflu4387/3 | - | dhpg=dpg=hpg-like specificity (1.804) | dab |
| PAO1 PvdI/1 | ser | ser=thr=ser/thr=dht=dhpg=dpg=hpg-like specificity (0.935) | ser |
| PAO1 PvdI/2 | - | asp=asn=glu=gln=aad-like specificity (1.744) | arg |
| PAO1 PvdI/3 | ser | ser=thr=ser/thr=dht=dhpg=dpg=hpg-like specificity (0.935) | ser |
| PAO1 PvdI/4 | - | dhb=sal-like specificity (1.178) | fOHOrn |
| PAO1 PvdJ/1 | - | asp=asn-like specificity (1.257) | lys |
| PAO1 PvdJ/2 | - | dhb=sal-like specificity (1.178) | fOHOrn |
| PAO1 PvdD/1 | thr | thr=dht-like specificity (1.248) | thr |
| PAO1 PvdD/2 | thr | thr=dht-like specificity (1.248) | thr |
| PAO1 PvdL/1 | glu | asp=asn=glu=gln=aad-like specificity (1.032) | glu |
| PAO1 PvdL/2 | lys | asp=asn=glu=gln=aad-like specificity (1.639) | tyr |
| PAO1 PvdL/3 | - | dhpg=dpg=hpg-like specificity (1.804) | dab |

^a^ Prediction according to specificity conferring code by Stachelhaus et al [37]. Amino acid designations are as per IUPAC convention. Hyphen denotes that no prediction was made for that module.

^b^ Prediction using transductive support vector machine (TSVM) method [36]. For each module, the highest scoring amino acid cluster prediction is shown. Clusters are based on amino acids with similar physico-chemical properties, and/or similar substrate binding pockets as defined by Rausch et al [36]. Additional amino acid designations: aad, 2-amino-adipic acid; abu, 2-amino-butyric acid; dab, 2,4-diaminobutyrate; dhb, 2,3-dihydroxy-benzoic acid; dhpg=dpg, 3,5-dihydroxy-phenyl-glycine; dht, dehydro-threonine; hpg, 4-hydroxy-phenyl-glycine; iva, isovaline; sal, salicylic acid.

^c^ Comparison of the SBW25 PVD peptide backbone with SBW25 NRPS substrate *in silico* predictions suggest *pvdI* and Pflu2544 are involved in the synthesis of SBW25 PVD.

^d^ The substrates of PAO1 PvdL were determined by Mossialos et al [21].
