# Supplementary material for: Genomic, genetic and structural analysis of pyoverdine-mediated iron acquisition in the plant growth-promoting bacterium Pseudomonas fluorescens SBW25
Source: BMC Microbiol. 2008 Jan 14;8:7. doi: 10.1186/1471-2180-8-7 (PMC2235872; doi:10.1186/1471-2180-8-7)
Supplement: Additional file 3 — List of putative siderophore receptors in P. fluorescens SBW25. The data list all putative siderophore receptors in the SBW25 genome, and whether these are adjacent to predicted sigma factor/anti-sigma factor gene pairs. [file 1471-2180-8-7-S3.doc]

**Additional File 3 – List of putative siderophore receptors in *P. fluorescens* SBW25**

| Pflu number^a^ | Product | Presence of sigma/anti-sigma factor genes^b^ |
| --- | --- | --- |
| 295 | putative iron-transport related export protein | - |
| 757 | putative TonB-dependent siderophore receptor | - |
| 1022 | putative ferric siderophore receptor | - |
| 1040 | putative *fecA* homologue | 1042/1041 |
| 1405 | putative haem utilisation protein | 1407/1406 |
| 2202 | putative TonB-dependent siderophore receptor | - |
| 2216 | putative enterobactin receptor | - |
| 2365 | putative TonB-dependent siderophore receptor | - |
| 2545 | *fpvA* - ferripyoverdine receptor precursor | *-* |
| 2562 | putative ferric siderophore receptor | - |
| 2598 | putative *bfrH* homologue | - |
| 2688 | putative ferric alcaligin siderophore receptor | - |
| 2948 | putative TonB-dependent siderophore receptor | - |
| 3378 | putative ferric alcaligin siderophore receptor | - |
| 3566 | putative *fcuA* - ferrichrome receptor protein | 3568/3567 |
| 3633 | putative *fauA* - ferric alcaligin siderophore receptor | 3631/3632 |
| 3643 | putative exogenous ferric siderophore receptor | - |
| 4093 | putative ferric siderophore receptor | - |
| 4968 | putative exported heme receptor protein | 4966/4967 |
| 5361 | putative ferripyoverdine receptor precursor | 5363/5362 |
| 5629 | putative outer membrane ferrichrome-iron receptor | 5627/5628 |
| 5798 | putative iron receptor exported protein | 5800/5799 |
| 5895 | putative TonB-dependent siderophore membrane receptor | - |
| 6132 | putative ferric siderophore receptor | 6130/6131 |

^a^ Genes were identified on the basis of hits to Protein Family (Pfam) accessions PF00593 (TonB-dependent receptor) and PF07715 (TonB-dependent receptor plug domain), and BLAST homology to siderophore receptor genes or genes involved in iron uptake.

^b^ Pflu numbers are listed for ORFs adjacent to receptor genes with homology to sigma/anti-sigma factor genes via hits to Pfam accessions PF04542 (Sigma-70 region) and PF04773 (FecR protein), respectively. Hyphens indicate no homology was detected.
